# Ocular Surface Features in Patients with Parkinson Disease on and off Treatment: A Narrative Review

**DOI:** 10.3390/life12122141

**Published:** 2022-12-19

**Authors:** Matilde Buzzi, Giuseppe Giannaccare, Michela Cennamo, Federico Bernabei, Pierre-Raphael Rothschild, Aldo Vagge, Vincenzo Scorcia, Rita Mencucci

**Affiliations:** 1 Department of Biomedical Sciences, Humanitas University, Pieve Emanuele, 20072 Milan, Italy; 2 Department of Ophthalmology, University Magna Græcia of Catanzaro, 88100 Catanzaro, Italy; 3 Eye Clinic, Department of Neurosciences, Psychology, Pharmacology and Child Health, University of Florence, 50134 Florence, Italy; 4 Service d’Ophtalmologie, Ophtalmopôle de Paris, Hôpital Cochin, AP-HP, F-75014 Paris, France; 5 Eye Clinic of Genoa, Policlinico San Martino, Department of Neuroscience, Rehabilitation, Ophthalmology, Genetics, Maternal and Child Health (DiNOGMI), University of Genoa, 16132 Genoa, Italy

**Keywords:** Parkinson’s disease, ocular surface, cornea, tears, dry eye, amantadine

## Abstract

Parkinson disease (PD) is a progressive, neurodegenerative disease of the central nervous system. Visual disturbance is one of the most frequent nonmotor abnormalities referred to by patients suffering from PD at early stages. Furthermore, ocular surface alterations including mainly dry eye and blink reduction represent another common finding in patients with PD. Tears of PD patients show specific alterations related to protein composition, and in vivo confocal microscopy has demonstrated profound changes in different corneal layers in this setting. These changes can be attributed not only to the disease itself, but also to the medications used for its management. In particular, signs of corneal toxicity, both at epithelial and endothelial level, are well described in the literature in PD patients receiving amantadine. Management of PD patients from the ophthalmologist’s side requires knowledge of the common, but often underdiagnosed, ocular surface alterations as well as of the signs of drug toxicity. Furthermore, ocular surface biomarkers can be useful for the early diagnosis of PD as well as for monitoring the degree of neural degeneration over time.

## 1. Introduction

Parkinson disease (PD) is a progressive, neurodegenerative disease of the central nervous system, particularly of the basal ganglia and dopaminergic pathways, characterized by resting tremor, muscular rigidity, bradykinesia, and postural instability. The disease also determines visual pathway changes since dopamine is contained in amacrine cells in the inner plexiform layer of the retina and plays multiple roles in the eye. In fact, it takes part in light adaptation, spatial contrast sensitivity, color discrimination, oculomotor control, and the photoreceptor renewal process, as well as probably is involved in the cyclic regulation of intraocular pressure and plays an anti-apoptotic role [1]. Therefore, it is not surprising that PD, characterized by progressive dopamine depletion due to dopaminergic neuronal death in the substantia nigra, can have a high detrimental impact on visual performance. As a matter of fact, visual disturbance is one of the most frequent nonmotor abnormalities referred to by patients suffering from PD [2]. Ocular changes described in patients with PD are impaired visual function (decreased color discrimination and contrast sensitivity, visuospatial deficit), visual hallucinations, altered eyelid movements (reduced spontaneous blink, blepharospasm, apraxia of lid opening), and abnormal eye movements (decreased saccades performance and adaptive modification of saccade amplitudes, deficit of memory-guided saccades, ocular microtremor) [3].

Ocular surface alterations represent other common findings in patients with PD and include mainly dry eye and reduced blink rate (BR) [3]. The examination of the cornea by means of in vivo confocal microscopy (IVCM), which allows an analysis of the cornea at a cellular level, has demonstrated profound changes in different corneal layers in PD patients. These changes were attributed not only to the disease itself, but also to the medications used for its control [4]. For instance, signs of corneal toxicity, both at epithelial and endothelial level, are well described in literature in PD patients receiving amantadine.

This review aims at comprehensively discussing ocular surface changes occurring in patients with PD related to both the underlying disease and the drugs used for its control.

## 2. Materials and Methods

The present review was conducted by a systematic computerized search using the following key words and their abbreviation in the electronic PubMed database: “tear” OR “cornea” OR “conjunctiva” OR “ocular surface” AND “Parkinson’s disease” OR “neurodegenerative diseases” OR “neurologic therapies” OR “dopaminergic agents” OR “amantadine”. No language restriction was applied and references of retrieved articles were scanned manually to identify additional studies.

The articles were considered eligible if their main topic was to investigate the effect of PD and its therapies on the ocular surface and cornea. Abstracts from conferences, letters, reviews, duplicate publications, and full texts without raw data available for retrieval were excluded. This article is based on previous studies and does not contain information from studies conducted on human participants or animals by any of the authors.

## 3. Results

In recent years, several studies have confirmed that the ocular surface picture is often altered in the setting of neurodegenerative diseases [2,5].

Patients suffering from PD present ocular surface alterations, such as a reduced BR, decreased corneal sensitivity, abnormal tear function and composition, and meibomian gland dysfunction (MGD). Ocular surface involvement in patients with PD has been demonstrated even in initial stages of the disease, in patients with early, untreated PD [3]. Additionally, patients with moderate to advanced disease, often on chronic medication, can suffer from ocular side effects of pharmacologic therapy [4].

### 3.1. Ocular Surface Parameters in PD

Patients with PD have some peculiar ocular surface changes related to the pathogenesis of the disease [6]. Ocular surface parameters have been studied in relation to the Hoehn and Yahr scale (H&Y), a commonly used system for PD progression ranging from 0 (no signs of disease) up to 5 depending on the increasing severity of the disease [7]. This scale is usually used together with the unified PD rating scale (UPDRS) that analyzes mental activity, daily activities, motor function, and treatment complications attributing a score ranging from 0 (no disability) up to 199 (total disability) [8].

#### 3.1.1. Simple Tests for Tear Film Characterization

A recent meta-analysis revealed that patients with PD, compared to healthy controls, may more likely present with dry eye disease, with a 61.1% prevalence of subjective ocular discomfort symptoms [9]. In fact, ocular surface disease index (OSDI) scores are reported to be higher in PD patients than in controls [1,10].

Among patients with PD, tear break-up time (TBUT) shows a notable decrease [9]: nervous system dysregulation associated with PD progression causes the alteration of aqueous, lipid, and mucin composition, leading to tear film instability. A significant decrease in Schirmer test values is also described in PD patients, confirming the theory that PD decreases both baseline and reflex lacrimation [9]. Also, corneal fluorescein staining, corneal-rose bengal staining and phenol red thread test are reported to be worsen in PD patients [11].

Bagheri et al. [12] reported that patients with H&Y stages 3–4 had reduced tear secretion compared to healthy controls while the difference was not significant in patients with lower H&Y stages (1–2). Tamer et al. [11] demonstrated that abnormalities in ocular surface parameters occurred at a higher rate in PD patients compared to healthy controls, and both Schirmer and TBUT reductions were inversely correlated with the H&Y score.

When considering tear flow, it is important to note that several systemic drugs used to treat PD including anticholinergic (antimuscarinic agents), anticholinergic-like (amantadine) and antidepressants may impair tear production [13]. Since the majority of PD patients studied in literature were treated with these medications, the observed reduction in tear secretion could have occurred due to the PD pathology itself, drug side effects, or both. Biousse et al. [3] were the first to evaluate ocular surface parameters with early, untreated disease and reported a decreased TBUT compared to healthy controls while Schirmer’s values were not significantly different.

According to Nowacka et al. [1], meibomian glands are significantly affected in PD patients compared to controls. The severity of MGD was graded on the scale proposed by Bron et al. [14]: from grade 0 in which all glands are clear of blockage, to grade 4 in which more than 50% of the glands have viscous secretions. The mean MGD grade in PD patients was 1.23 ± 1.36, while 0.90 ± 1.16 in the control group [1]. These results are consistent with previous studies [11,12,15]; furthermore, other studies [3,11] suggest that PD patients can also have deficiency in the tear film mucin layer. In a recent study, Blinchevsky and coworkers [16] characterized meibum tear lipids in donors with PD, compared to a control group: changes in meibum lipid composition and conformation could contribute to and increase the susceptibility of dry eye in patients with PD.

Dry eye symptoms can also be aggravated by blepharitis, which is common in PD patients, most likely secondary to seborrhoea. In fact, seborrheic blepharitis was diagnosed in 17.86% of eyes of PD patients while in 3.06% eyes of control cases [1]. According to Biousse [3], although 75% of PD patients had blepharitis, this percentage did not differ from the control population.

#### 3.1.2. High-Tech Imaging Devices

Several studies demonstrated that the BR is significantly lower in PD patients compared to controls [3,10,11,17,18] and concomitant signs and symptoms of dry eye were present. The reduced BR could be associated with reduced nigrocollicular pathway activity [19] and is considered a sign that supports PD diagnosis. In a study examining the BR in patients with PD in different daily situations (e.g., while being interviewed, watching a video, and reading a book), this parameter was significantly lower in the PD patients compared to controls; however, no correlation was found between BR and disease severity [20]. Conversely, other studies have reported a negative correlation between the H&Y scores and BR in patients with PD [11,21]. Agostino and coworkers [22] investigated the kinematic blinking features in patients with PD, off and on dopaminergic treatment. Patients off therapy pause longer than controls during voluntary blinking and the spontaneous BR tends to be lower, as an effect of bradykinesia. Dopaminergic treatment shortens the pause during voluntary blinking and increases the spontaneous BR.

The reduced BR together with decreased tear production and poor tear quality can result in reduced central corneal thickness (CCT). Aksoy et al. [18] analyzed CCT in a group of patients with PD: this parameter was significantly lower in PD compared to controls. In addition, significant decrease in CCT was registered in the patient group as the H&Y score increased. According to Ulusoy et al. [17] pachymetric measurements, Bowman layer and stromal thickness values of PD patients were significantly lower than those of controls and negatively correlated with disease duration and severity. On the contrary, the values of TBUT and Schirmer test were significantly positively correlated with pachymetric measurements and stromal thickness.

Given that BR may be influenced by corneal nerves, these structures have been investigated in patients with PD. Several studies have found corneal changes using IVCM in PD patients [23,24,25,26,27,28,29]. Arrigo and coworkers [29] described corneal innervation and trigeminal alterations in 3 drug-naive patients with PD, comparing them to controls. Strong differences were found for deep nerve tortuosity and the number of beadings, revealing significant alterations to the level of both deep and sub-basal corneal plexa in patients with PD. Beadings were reported at the level of the deep corneal nerve plexus with a higher number compared to controls. Furthermore, the tortuosity index was significantly higher in both eyes of all patients and a significant reduction (approximately 50%) of corneal sub-basal nerve plexus density was demonstrated in patients with moderate to severe PD [27]. Several authors found a significantly reduced corneal nerve fiber density (CNFD) with increased corneal nerve branch density (CNBD) and corneal nerve fiber length (CNFL) in patients treated for PD compared with controls [23,24,25]. Podgorny et al. [30] found that early untreated PD patients had significantly reduced CNBD and CNFL (but not CNFD) compared to controls. According to Lim et al. [24], CNFD, CNBD, CNFL and corneal total branch density were significantly lower in PD patients at every stage of the disease compared to controls.

No association between alteration of corneal nerve metrics and PD duration was demonstrated [23,24].

Regarding the correlation between corneal nerves and cognitive impairment, several studies showed that decreased corneal sub-basal nerve density was strongly related to cognitive impairment [25,26,27]. Che and colleagues demonstrated that CNBD and the CNBD/CNFD ratio was higher in PD patients with normal cognitive function compared to controls, and CNFD was positively correlated with the Montreal cognitive assessment score, one of the most used scales to detect cognitive impairment [25]. In addition, Misra et al. [27] demonstrated a significant positive correlation between Addenbrooke’s cognitive examination revised scores, used to assess cognitive function, and sub-basal corneal nerve density. On the contrary, according to Lim et al. [24], there was no correlation between corneal nerve parameters and cognitive function in PD patients.

No relationship between sub-basal corneal nerve density and autonomic dysfunction, assessed using the Survey of Autonomic Symptoms score, was found [27].

Regarding the relationship between corneal nerves and motor symptoms in PD patients, CNBD, CNFD, and CNFL were negatively associated with UPDRS part III (motor examination) and total UPDRS scores [23,25]. In Lim et al.’s study, the UPDRS part III score over 12 months was significantly higher in patients with a CNFD in the lowest quartile compared to the highest one at baseline [31]. Furthermore, a lower CNFL predicted progressive worsening of UPDRS-III over 1 year in patients with PD [31]. Moreover, CNFD, CNBD, CNFL, and the CNBD/CNFD ratio resulted in being increasingly altered with higher Hoehn and Yahr stage [25,32]. A strong correlation between BR and corneal sub-basal nerve density has been described in research by Reddy et al. [28]. However, in another study, the corneal sub-basal nerve density and BR presented no statistically significant association [27].

When the IVCM corneal parameters of the clinically more affected side were compared with those of the less affected side in asymmetry PD patients, two different studies found no significant difference in the sub-basal corneal nerve plexus density values between the two sides [23,27]. Conversely, corneal sensitivity was found to differ significantly between the two sides in asymmetrical cases.

Currently, the effect of PD medication on peripheral neuropathy is still unclear. In a study by Andreasson and colleagues [26], a correlation between the duration of levodopa therapy and CNBD was found. Other studies found no significant correlation between CNBD and CNFL and cumulative levodopa dose [23] or between CNFD and levodopa equivalent daily dose [25].

According to recent research, amantadine may affect corneal nerve fibers. Daggumilli et al. [33] showed that corneal sub-basal nerve fiber layer significantly decreased in patients under therapy with PD compared to controls at 1 year. Furthermore, corneal sub-basal nerve fiber layer was lower in PD amantadine compared to a PD amantadine naive group [33].

#### 3.1.3. Biochemical Investigation of Tear Film

Tears are a known source of biomarkers for both ocular and systemic diseases. PD patient basal and reflex tears have been assayed for disease biomarkers. A mass spectrometry study of tear proteome in PD patients demonstrated that core networks of proteins involved in immune response, lipid metabolism, and oxidative stress are distinctly regulated in such patients [34]. Furthermore, the TNF-α levels, measured by a multiplex immunohead assay, resulted in being significantly higher in PD patients, suggesting that neuroinflammation plays a role in its pathogenesis [35]. Tear TNF-α levels, however, were not related to the duration and severity of the disease [35].

Hamm-Alvarez and colleagues have studied the expression of α-synuclein in tears of PD patients [36]. Alpha-synuclein is a precursor to the Lewy bodies, aggregates found in the substantia nigra of PD patients, but also throughout the central and peripheral nervous systems as well as in other organ systems. Alpha-synuclein exists both as an unfolded monomer and folded oligomers. In PD, this balance is altered, leading to the formation of α-Synuclein oligomers (α-SynOligo) that form Lewy bodies. In basal tears from PD patients α-SynTotal is decreased, whereas α-SynOligo and the ratio of α-SynOligo/α-SynTotal are increased [37]. This difference is even more pronounced in reflex than in basal tears: in PD patient reflex tears α-SynOligo, CCL2 (a protein implicated in PD) and lactoferrin are significantly elevated [36].

More recently, Acera and coworkers [38] analyzed the tear proteome profile of patients with idiopathic PD by nano-liquid chromatography–mass spectrometry, while assessing their neurological impairment. These findings revealed that certain proteins, mainly involved in lysosomal function, were up-regulated in the tears of PD patients and may be related to an aggressive PD phenotype.

Bogdanov et al. [39] analyzed the change in the tear concentration of selected catecholamines (dopamine, noradrenaline, adrenaline) and α-2-macroglobulin, a protease inhibitor involved in the pathogenesis of PD. It was shown that tears in PD patients are characterized by an increased level of noradrenaline mainly on the ipsilateral side of pronounced motor symptoms, a decreased level of adrenaline on both sides, and an increased α-2-macroglobulin activity on both sides compared to controls. This study showed that adrenaline, noradrenaline, and the analysis of α -2-macroglobulin activity have the greatest potential as biomarkers (81.2%, 88.9%, and 92%, respectively).

Another study investigating the changes in catecholamine content in tears in PD subjects [40] found that the concentration of norepinephrine increased twice, the dopamine content increased by about 50% (only in the ipsilateral side), and the concentration of epinephrine on both sides decreased twice. These results were confirmed by studies on an animal model of PD, where an increase in the content of norepinephrine in tears was also observed [41]. Therefore, catecholamines may have a high potential to serve as marker of early clinical stages of PD.

### 3.2. Influence of PD Therapies on Cornea

Various medications employed for neurological diseases have been associated with ocular side effects. Drugs can be deposited at various sites of the eye, and tissues with high metabolic rates, such as retina and optic nerve, are the most vulnerable ones [4]. Less frequently, the cornea may be negatively affected by neurological therapies as well. Among drugs commonly used in PD therapy, amantadine is the only one that has been shown to cause ocular surface alterations.

Amantadine is a non-competitive antagonist of the nmethyl-D-aspartate receptor. Amantadine was first approved for influenza prophylaxis; subsequently, it was found that it was able to also address symptoms of PD. Today, it is widely used in the management of PD, tardive dyskinesia, levodopa-induced dyskinesia, and fatigue in subjects with multiple sclerosis. While the most common adverse effects are associated with central nervous system function (anxiety, agitation, and modulation of epileptic or psychiatric symptoms), this drug can induce adverse corneal reactions as well, such as superficial punctuate keratitis, punctuate subepithelial opacification, epithelial edema, and endothelial alterations that lead to stromal edema.

#### 3.2.1. Epitheliopathy

**Figure 1:**
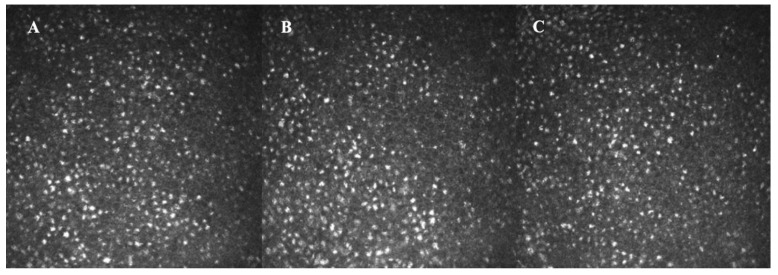
In vivo confocal microscopy findings showing highly reflective round shaped epithelial deposits induced by amantadine in a patient of 73 years who used the drug for 5 months for the treatment of Parkinson disease (**A**–**C**).

Amantadine is associated with corneal deposits and keratitis, being able to determine diffuse punctate subepithelial corneal opacities, more prominent in the infero-nasal region, occasionally with a superficial punctate keratopathy, corneal epithelial edema, and reduced vision [42]. Usually, the corneal impairment starts 1–2 weeks after treatment initiation. After drug interruption, keratopathy disappeared in the totality of cases in a few weeks. A possible explanation is that amantadine may be secreted in the tear film, thereby causing corneal deposits (Figure 1).

In the literature, several reports describe ocular side effects due to amantadine. Pearlman et al. [43] reported transient visual acuity impairment in a 67-year-old patient who received oral amantadine hydrochloride for 3 weeks that improved after discontinuation of the treatment. According to Blanchard, a 64-year-old patient who received oral amantadine hydrochloride for 19 days had transient blurred vision associated with corneal epithelial edema that recovered rapidly after withdrawal of the drug [44]. Nogaki et al. described a case of possible amantadine hydrochloride-induced impaired visual acuity and corneal damage (superficial punctate keratitis and corneal abrasion) in a patient with parkinsonism due to cerebral infarction [45]. The symptoms started after about 3 weeks of treatment and disappeared with drug withdrawn, but a recurrence was noted when treatment was resumed. More recently, Yoshinaka described a case of an 81-year-old woman, treated with amantadine for 9 years, who presented with impaired vision, showing opacities in the corneal epithelial corneal layer [46]. On IVCM, there were highly reflective deposits in corneal epithelial cells, without pathological findings in stroma and endothelium. Two months after treatment discontinuation, corneal opacities disappeared, and visual acuity was restored.

#### 3.2.2. Corneal Edema

Amantadine can cause corneal edema that starts a few months to several years after treatment initiation. In the literature, several reports describe reversible amantadine-induced corneal edema in patients chronically taking this medication, for PD or other diseases [47,48,49,50,51,52]. In most of the cases described, the duration of use of amantadine ranged from 2 months to 6 years before the onset of this complication. Cases may be asymmetrically bilateral. The onset is acute, and the clinical presentation is of painless, progressive visual loss. Corneal edema often appears posteriorly and centrally and, in some cases, progresses to diffuse edema. No signs of ocular inflammation are present. Weeks to months after discontinuation of the drug, the corneal edema progressively resolves, and visual acuity returns to baseline in most cases. On the contrary, Jeng [51] described a case of prolonged corneal edema that became irreversible. Histopathologic analysis of the cornea buttons showed significant loss of endothelial cells that required corneal transplantation. The contralateral eye of the same patient, which had been previously transplanted, developed edema in the grafted cornea as well.

In a study including 13,137 patients receiving amantadine over 2 years, 36 were newly diagnosed with corneal edema (0.27% of total cases); 73% of these cases occurred in females [53].

Early diagnosis and interruption of amantadine maximizes the likelihood of achieving corneal clarity, avoiding the need for corneal transplantation. However, when amantadine cannot be discontinued because no alternative therapy is available, resuming amantadine with the addition of corticosteroids may help to preserve corneal endothelial cell density (ECD) [48,51].

**Figure 2:**
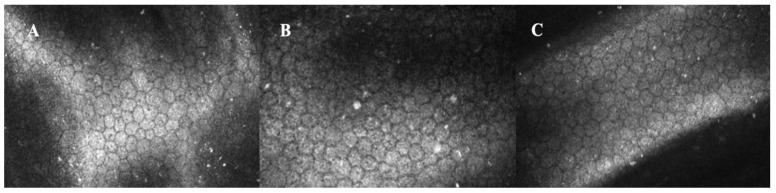
In vivo confocal microscopy of the cornea in a patient of 69 years who used amantadine for 2 years to treat Parkinson disease. The scans show decreased density and increased pleomorphism and polymegathism of corneal endothelial cells; furthermore, highly reflective deposits are present at this level (**A**–**C**).

The pathophysiological mechanism of transient amantadine-induced edema is poorly understood; however, based on specular microscopy and histopathologic studies, endothelial cell death appears to be induced or accelerated, with a resulting decreased ECD (Figure 2) [48,49]. Chang and colleagues [54] demonstrated that patients taking amantadine had significantly lower ECD, lower hexagonality, and greater coefficient of variation compared with an age-matched control group. Daggumilli et al. [33] obtained the same results in amantadine patients with PD compared with amantadine naive patients with PD and controls, respectively. Dudle’s experiments [55] on bovine corneas demonstrated that amantadine increased endothelial cell volume without increasing apoptotic cell death. Prolonged cell volume dysregulation may then lead to cell death and account for the decreased corneal ECD noted in patients on amantadine therapy. Their studies concluded that amantadine’s effects on corneal endothelium are mediated via K+ channels. In fact, amantadine is also a K+-channel blocker. The transient effect on short-circuit current support the reversibility of amantadine-induced corneal edema.

The effects of duration of amantadine treatment are unclear and it is possible that amantadine toxicity is not related to the cumulative dose. In a Taiwan-based nationwide cohort study of patients with PD, a hazard ratio of 1.79 for corneal edema with amantadine use increased to 2.05 for those using moderate doses (2000–4000 mg) and 2.84 for those using high doses (>4000 mg) [56]. Daggumilli et al. [33] demonstrated a greater change in endothelial parameters in patients who were on 400 mg amantadine compared to 200 or 300 mg. In fact, longer duration and higher cumulative dose of amantadine therapy seems to determine a greater reduction in ECD compared to age-matched controls [54]. Lee and coworkers [56] studied the effects of amantadine HCl on cell growth, proliferation, and apoptosis in bovine corneal endothelial cells, and examined in vitro endothelial permeability. Results showed that low doses of amantadine HCl do not affect cell growth, whereas higher doses inhibit it, induce apoptosis, increase sub-G1 phase growth arrest, cause DNA damage, and induce endothelial hyperpermeability in bovine cornea endothelial cells; additionally, Lee also demonstrated that amantadine HCl attenuates the proliferation and arrests cell cycle at G1 phase in bovine corneal endothelial cells.

## 4. Discussion

Patients affected by PD often suffer from ocular surface alterations. According to the literature, more than 60% of patients with PD exhibit subjective ocular discomfort symptoms [9]. Additionally, PD patients exhibit significantly decreased BR, corneal thickness, TBUT, and tear productions compared to controls. These findings highlight the possibility of the comorbid occurrence of dry eye disease with PD, emphasizing the need for screening and appropriate interventions for clinicians managing patients with PD. These patients may benefit from prompt ophthalmologic examinations, complete of ocular surface tests (TBUT, Schirmer’s test, Fluorescein staining, OSDI), at the time of the diagnosis and along the course of the disease. By recognizing the common—but often underdiagnosed—ocular surface impairment in this setting, physicians can prescribe a proper treatment in order to improve patients’ symptoms. If possible, medications that interact with tear secretion and accommodation should be avoided; on the contrary, the use of artificial tears to provide adequate ocular surface lubrication should be encouraged. Punctual occlusion should be considered in patients with severe dry eye. Furthermore, ophthalmologists should remember the possible comorbid presence of seborrheic blepharitis and MGD, which require eyelid hygiene.

Moreover, ocular surface can be used as a window to the PD, and novel ocular biomarkers, helpful for an early diagnosis of the underlying disease, can be developed and used. In particular, thanks to the lack of invasiveness of tear collection and the relatively simple composition of tears compared to serum and plasma, they can represent a rapid and cheap method for the detection of specific proteins that correlate with PD. Recent studies have identified some PD biomarkers present in tears, such as α-synTotal and α-synOligo, catecholamines, α-2-macroglobulin, and other proteins involved in immune response, lipid metabolism, and oxidative stress. However, the current state of knowledge is insufficient to clearly indicate certain biomarkers of the diseases, as many of the current studies are of a pilot nature and larger study group analyses are needed. In the future, this research may allow the development of commercial screening tests focused on a rapid, non-invasive, and early PD diagnosis.

IVCM parameters may act as a biomarker for neurodegeneration in PD as well. Corneal innervation changes occur early in PD patients and IVCM analysis might provide biomarkers for early PD diagnosis when no other obvious changes can be found [30]. In fact, the cornea is the most densely innervated human tissue, and changes in corneal nerve fibers precede the ones in cutaneous nerve fibers; furthermore, IVCM may also be an interesting noninvasive and reproducible tool to monitor the efficacy of drugs used in this setting.

Finally, ophthalmologists and neurologists should be aware of the potential toxicity of PD medications on the cornea. In particular, amantadine has been shown to cause ocular surface alterations. Before starting such therapy, baseline evaluation of the corneal endothelium should be performed, to assess the patient’s risk–benefit ratio and target the neurological therapy on this basis. Regular assessment of endothelial cells is recommended in patients at risk when amantadine has been administered long term and/or at high doses. If visual acuity is suddenly impaired in these patients, the status of the cornea should be examined and in case of corneal alterations, drug discontinuation should be considered, whenever possible.

## 5. Conclusions

Visual impairment is a possible sensory symptom in PD, both secondary to the disease itself and its chronic medications. Ocular surface changes, involving lids, tears, conjunctiva, and cornea, are responsible for most of the ocular complaints of patients affected by PD. Therefore, clinical examination of PD patients by ophthalmologists requires awareness of the common, often underdiagnosed, ocular surface alterations. By addressing the specific sources of patients’ complaints and offering some measures of intervention, a significant improvement in a patient’s quality of life may be obtained. Finally, since neurodegenerative disorders are often diagnosed when extensive neuronal damage has already occurred, some ocular surface biomarkers can be useful for the early diagnosis of the underlying disease as well as for monitoring the degree of neural degeneration over time. Recent studies have identified PD biomarkers present in tears and IVCM analysis might allow early PD diagnosis, as corneal innervation changes occur early in PD patients. Further research is necessary for the development of screening tools focused on a fast and early PD diagnosis.

## Data Availability

Not applicable.
